# Relationships reduce risks for child maltreatment: Results of an experimental trial of Infant Mental Health Home Visiting

**DOI:** 10.3389/fpsyt.2023.979740

**Published:** 2023-02-28

**Authors:** Megan M. Julian, Jessica Riggs, Kristyn Wong, Jamie M. Lawler, Holly E. Brophy-Herb, Julie Ribaudo, Ann Stacks, Jennifer M. Jester, Jerrica Pitzen, Katherine L. Rosenblum, Maria Muzik, on behalf of The Michigan Collaborative for Infant Mental Health Research (MCIMHR)

**Affiliations:** ^1^Department of Psychiatry, University of Michigan, Ann Arbor, MI, United States; ^2^Department of Psychology, Eastern Michigan University, Ypsilanti, MI, United States; ^3^Department of Human Development and Family Studies, Michigan State University, East Lansing, MI, United States; ^4^School of Social Work, University of Michigan, Ann Arbor, MI, United States; ^5^School of Social Work, Wayne State University, Detroit, MI, United States; ^6^Merrill Palmer Skillman Institute, Wayne State University, Detroit, MI, United States; ^7^Department of Obstetrics and Gynecology, University of Michigan, Ann Arbor, MI, United States

**Keywords:** child maltreatment risk, home visiting, infant mental health, Brief Child Abuse Potential Inventory, parenting, mother-infant dyads, child abuse – prevention and control

## Abstract

**Background:**

Research examining the effectiveness of home visiting programs that reduce child maltreatment or associated risks yield mixed findings; some find positive significant impacts on maltreatment, whereas others find small to no effects. The Michigan Model of Infant Mental Health Home Visiting (IMH-HV) is a manualized, needs-driven, relationship-focused, home-based intervention service that significantly impacts maternal and child outcomes; the effect of this intervention on child maltreatment has not been sufficiently evaluated.

**Objective:**

The current study examined associations between treatment and dosage of IMH-HV and child abuse potential in a longitudinal, randomized controlled trial (RCT).

**Participants and setting:**

Participants included 66 mother-infant dyads (Mother *M* age = 31.93 years at baseline; child *M* age = 11.22 months at baseline) who received up to 1 year of IMH-HV treatment (*Mdn* = 32 visits) or no IMH-HV treatment during the study period.

**Methods:**

Mothers completed a battery of assessments including the Brief Child Abuse Potential Inventory (BCAP) at baseline and at the 12-month follow-up assessment.

**Results:**

Regression analyses indicated that after controlling for baseline BCAP scores, those who received any IMH-HV treatment had lower 12-month BCAP scores compared to those who received no treatment. Additionally, participation in more visits was associated with lower child abuse potential at 12 months, and a reduced likelihood of scoring in the risk range.

**Conclusion:**

Findings suggest that greater participation in IMH-HV is associated with decreased risk for child maltreatment 1 year after initiating treatment. IMH-HV promotes parent-clinician therapeutic alliance and provides infant-parent psychotherapy which differentiate it from traditional home visiting programs.

## Introduction

Identifying ways to prevent maltreatment is critical in early childhood (1). During the first years of life, maltreatment can have significant and lasting effects on physical and mental health (2, 3) and later academic performance (4). Prevention and intervention efforts target known risk factors for childhood maltreatment, including demographic characteristics related to systemic inequities, encompassing socioeconomic factors, parents’ mental health, substance use, and their own history of childhood adversity (5–7). There is exponentially greater risk conferred for child maltreatment as risk factors accumulate (8). Therefore, the most effective interventions are likely to be those that address multiple risk factors and intergenerational patterns of risk while also focusing on improving parent–child relationships.

For many years, home visiting interventions supporting mothers, infants, and young children’s wellbeing have sought to ameliorate the risks associated with parenting in the context of poverty and other psychosocial risks such as low education and mental health symptoms (9, 10). Although some home visiting programs find positive effects on reducing child maltreatment [e.g., (1, 10, 11)], effects are often small (9) or non-significant (12, 13). These findings make it difficult to determine the effectiveness of home visiting programs in reducing child maltreatment or associated risks. Home visiting programs that improve parental reflective functioning and parenting behavior, while addressing parents’ histories of trauma, may be particularly effective [see (14, 15)].

Infant Mental Health Home Visiting (IMH-HV) was developed in Michigan by Fraiberg et al. (16). IMH-HV is a home visiting program that focuses on supporting infants, young children (typically pregnancy to child age 36 months), and their families who are at heightened risk for poor outcomes due to parent or child risk factors [for a detailed description of IMH-HV, see: (17, 18)]. IMH-HV services utilize Medicaid funding, and families can self-refer or be referred by a professional (e.g., pediatrician, child protection worker). In Michigan, IMH-HV is a manualized model (19) which provides flexible, needs-driven, relationship-focused, home-based psychotherapeutic services. As services are tailored to a family’s needs, IMH-HV may function as either preventative intervention services or as psychotherapeutic treatment, with mental health diagnoses given when indicated. Families receive services designed to improve the parent-infant relationship and both the infant’s and parent’s mental health through therapeutic work focused on the dyad. At weekly home visits, a master’s level mental health clinician (e.g., social worker, masters level psychologist) flexibly utilizes numerous IMH-HV intervention strategies (e.g., developmental guidance, infant-parent psychotherapy, provision of emotional and concrete support) to address the complex needs of each family (20, 21). IMH-HV is distinguished from other home visiting programs by its strong focus on fostering supportive relationships between the clinician and parent, and between parent and infant, and its provision of emotional and psychotherapeutic support in addition to concrete support. Although not required, nearly all parents receive parent-infant psychotherapy in the home visiting process. IMH-HV providers also attend to the impacts of systemic oppression on families through advocacy and emotional support. IMH-HV clinicians receive regular reflective supervision, which provides clinicians with a nurturing environment that enables them to better provide a safe holding environment in their relationships with the families they work with. The identification of parental goals emerge from working together and often includes supporting their children’s development and enhancing the parent–child relationship. Of particular focus in the IMH-HV model is attending to the nuances of parent-infant interaction, believing that the infant can often “lead” the provider and parent to a deeper understanding of the strengths and vulnerabilities within the relationship (18). Although there is not an expected length or dose of service, some research suggests that at least 6 months of service may be helpful in promoting more optimal outcomes (22).

IMH-HV is currently delivered to more than 1,700 parent-infant/toddler dyads in Michigan, primarily through the community mental health system. The Michigan Model of IMH-HV has been tested in a community-based open trial and is effective at decreasing harsh parenting and maltreatment risk (23), improving maternal sensitivity (24), and improving reflective functioning (25). In the context of an infant-toddler Court Team, IMH-HV was effective at improving parent–child interaction, parental reflective functioning, and child development and was associated with high rates of reunification (26, 27). However, to conclusively attribute effects to the intervention, the current study reports on maltreatment risk using a comparison of those receiving IMH-HV services versus a control condition.

## Methods

### Study design/procedure

Data come from a longitudinal, experimental trial of IMH-HV (trial registration: NCT03175796). This study utilizes data from the baseline and 12-month follow-up assessments, which took place between October 2017 and September 2019.

Participants were recruited through a research registry of women who had recently given birth and were broadly interested in participating in research, from flyers posted in the community, or were referred from providers. The intervention program was described as a convenient in-home support service offered to parents and their young children that could include guidance and support on infant/toddler growth and development education, parenting techniques for overcoming challenging child behaviors, and emotional support and counseling. Study eligibility requirements were chosen so study participants broadly represented Medicaid eligible individuals who receive IMH-HV services in the community. Study eligibility was determined on screening phone calls to potential participants by endorsement of two of the following: eligibility for public benefits, report of parenting challenges and/or perceptions of their child as difficult, endorsement of high experiences of adversity during childhood (ACEs), and a screening score indicating possible depression. Additional requirements were maternal age ≥ 18 years, absence of symptoms of substance use disorder or psychosis, child age ≤ 24 months and legal custody of the child. Once enrolled, participants were randomly assigned to either treatment (offered up to 1 year of IMH-HV services) or control (offered a list of community resources) conditions using *a priori* urn randomization procedures; this technique ensured equal distribution of maternal ACEs, depression, and income across conditions. Participants received incentive payments (up to $435 for all study visits, biological samples, and questionnaires) for participation in this study. Participants who were randomized to the control group were provided with contact information for a state-provided free service that connects residents with health and human service agencies and resources. This study underwent IRB approval (ClinicalTrials.gov ID: NCT03175796. University of Michigan Medical School Institutional Review Board ID HUM00124224).

### Participants

**Figure 1:**
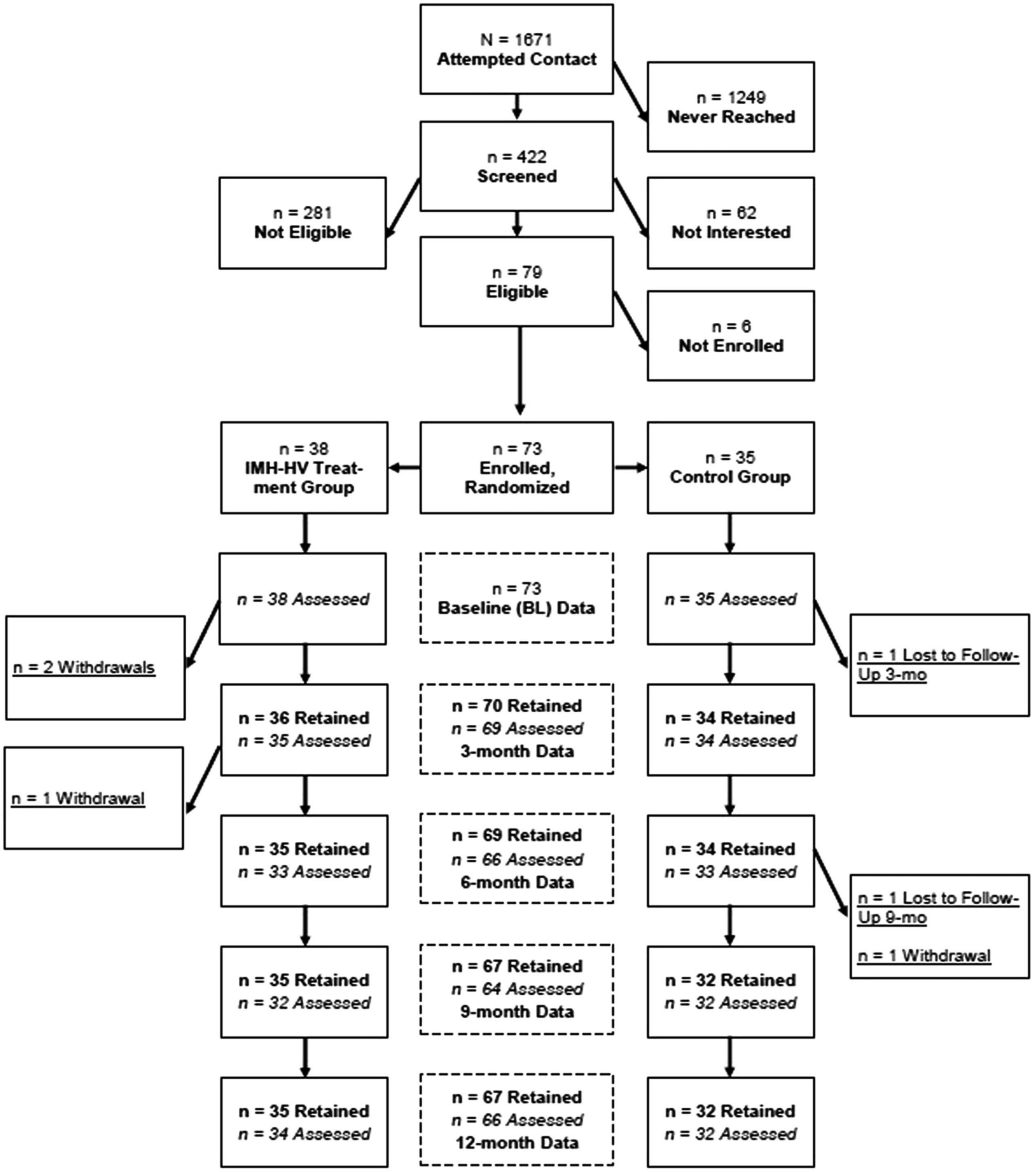
Consort diagram.

Participants were 66 mother-infant/toddler dyads who had 12-month follow-up assessment data available. Retention rates across the 12-month study period were high (90.41%; See consort diagram in Figure 1), and there were no significant demographic differences between those retained in the study and those lost to follow-up, suggesting no differential attrition. At both the baseline and 12-month follow-up assessments, data collection occurred in the home. During these visits, mothers completed measures including self-report questionnaires related to their own mental health, child social–emotional well-being/development, parenting, life events, and demographic information.

Maternal age at baseline ranged from 19.9 to 44.1 years (*M* = 31.93; *SD* = 5.57), and child age ranged from 0 to 23.8 months (*M* = 11.2; *SD* = 7.11; 7 mothers were in their third trimester of pregnancy at the time of study enrollment). Race and ethnicity reflected diversity within the sample (see Table 1).

**Table 1:** Sample demographic data (*n* = 66).

At study enrollment	Range		Mean (SD)
Child age (months)	0–24		11.22 (7.11)
Mother age (years)	19–44		31.91 (5.69)
Number of children (≤ 18 years) in the home	0–6		2.02 (1.25)
*Participant demographics*	% (*n*)	% (*n*)	
Race^1^	Parent	Child	
White	69.69% (46)	72.73% (48)	
African-American/Black	31.82% (21)	34.85% (23)	
Another race	5.48% (4)	12.12% (8)	
Ethnicity			
Hispanic or latinx	7.58% (5)	12.12% (8)	
Arab or Arab/American	3.03% (2)	4.55% (3)	
Parent education – High school or less	15.16% (10)		
Household income < $20,000 annually	24.24% (16)		
Currently receiving WIC	51.52% (34)		
Currently receiving medicaid/medicare	42.42% (28)		
*Study inclusion criteria*			
Screened eligible for government assistance^2^	63.0% (46)		
Probable maternal depression^3^	41.1% (30)		
Maternal history of childhood adversity^4^	67.1% (49)		
Endorsement of difficulty with parenting^5^	69.9% (51)		

^1^Percents do not add up to 100, as participants could select all racial and ethnic identities that applied. ^2^Eligible for Government Assistance = per-person household income < state threshold for eligibility for government services. ^3^Probable Maternal Depression = PHQ-9 score ≥ 10 (42). ^4^Maternal History of Childhood Adversity = Adverse Childhood Experiences (ACE) score ≥ 3 (43). ^5^Endorsement of Difficulty with Parenting = answer of “yes” when asked if parenting their child is difficult.

### Measures

#### Demographic information

Mothers completed self-report questionnaires to provide information on demographic data (see Table 1). Demographic information for this study comes from the baseline assessment, except for some child-specific demographic data (e.g., child sex) that were not available until later assessments for participants who were pregnant at enrollment.

#### Brief child abuse potential inventory

Potential to engage in child abuse was assessed using the Brief Child Abuse Potential Inventory [BCAP; (28)]. The BCAP is a 34-item measure comprised of items from the 160-item Child Abuse Potential Inventory (29). The BCAP is a caregiver-report questionnaire that measures endorsement of qualities known to increase the likelihood of child maltreatment. Constructs include caregiver emotional distress, rigidity, and social isolation. Mothers report agreement (1) or disagreement (0) with each item; some items are reverse-scored. BCAP responses result in two scales: the 9-item Validity scale, which screens for random responding and impression management (i.e., “faking good”), and the 25-item Abuse Risk scale, which measures risk of engagement in child maltreatment. Items on the Abuse Risk scale include items like “Children should be quiet and listen,” “Other people have made my life hard,” and “I often feel very alone.”

The Abuse Risk score may range from 0 to 25, with higher scores indicating a greater risk of engaging in child maltreatment. Cut-off scores of “9” and “12” are used to predict those at risk of engaging in child abuse (28); in this study we utilized the more conservative cut-off score of “9” to identify a risk cut-off. Participant scores were screened for invalid responses using the Validity scale. In our sample, 23 responses (34.8%) were above the suggested validity cut-off at baseline, and 22 (33.3%) were above the suggested validity cut-off at the 12-month time point; this is in line with the standardization sample which had 29.9% invalid responses (28). In order to maximize the sample size, results are presented with the full sample, but any changes to results with the reduced sample (removing responses above the suggested validity cut-off) are noted. Among this sample, reliability on the Abuse Risk scale was excellent (baseline *α* = 0.85; 12-month follow-up *α* = 0.86).

### Treatment

Thirty-eight mothers (57.6%) were randomly assigned to the treatment group, 33 of whom attended at least one session, five (13%) received no IMH-HV sessions. While all families assigned to treatment were approached by the clinician to begin treatment, five families did not follow through with attending any sessions. Of those assigned to treatment, a majority received multiple sessions of IMH-HV across the one-year treatment period. Number of sessions was determined by parents’ availability and need, and achievement of mutually agreed upon treatment goals (i.e., some families discontinued treatment before 1 year if all treatment goals were met). For this study, treatment group was used as a categorical variable, with those who *received* treatment (1 home visit or more) considered part of the treatment group, and those who *did not receive* treatment considered part of the control group; intent-to-treat analyses were not used due to the high number of participants assigned to the treatment group who did not receive any treatment. The treatment and control group did not differ in terms of demographic characteristics, including parent/child age, race/ethnicity, family income, or educational attainment. For those in the treatment condition, the median number of IMH-HV sessions received was 32 (*M* = 26.03, *SD* = 13.96).

### Data preparation and analytic plan

BCAP data were available for 66 parent-infant/toddler dyads from the baseline and 12-month follow-up. All data for the BCAP, demographics, and treatment variables were complete, and no data estimation was required. Analyses were conducted using SPSS Version 27.0.

Descriptive statistics were used to examine sample characteristics and to evaluate data normality. Linear regression models were utilized to examine associations between BCAP scores and treatment variables. Chi-square analyses were used to evaluate BCAP risk categorization by treatment group.

### Power analysis

To estimate power for this study, we used data from a previously published observational study in which all subjects received some level of treatment (23). To simulate an RCT, we separated the observational study participants into two groups, with the lowest 25% of treatment in the “control” group and the others in the “treatment” group. Due to higher power in an experimental study, we assumed that the current RCT would have an R-squared twice as high as the observational study and a lower residual variance (85% of the residual variance in the observational study). These estimates resulted in an effect size of 0.133 for the treatment effect. Using G*Power, we found that for the given effect size with alpha = 0.05 and power = 0.8, we would need a sample size of 61. Therefore, our current study with a sample size of 66 will have power greater than 0.8 to detect the effect of 0.133 or greater.

## Results

### Preliminary analyses

Correlations between treatment variables, child abuse potential variables, and baseline sample characteristic variables (Table 2) revealed that mothers with higher education received more IMH-HV sessions with a small effect size. Child abuse potential at baseline and 12 months was associated with lower maternal education with a small to medium effect size. Mothers with higher ACE scores had higher child abuse potential at baseline (medium effect size) and 12 months (small effect size). Higher child abuse potential scores at baseline and 12 months were associated with lower household income, single marital status, lower maternal age, and non-White race or ethnicity with small effect sizes in this sample. Many of the items on the child abuse potential measure refer to experiences that families who are non-White, low income, and/or experiencing systemic oppression are more likely to encounter because of historical, intentional, and structural racism, and do not indicate an association between demographic characteristics and child abuse propensity. Examples of such items include: “I sometimes worry that I will not have enough to eat,” and “People sometimes take advantage of me.”

**Table 2:** Correlations and descriptive statistics for key study variables.

	Treatment		Child abuse potential				Baseline sample characteristics						
	Treatment received	Number of IMH-HV visits	Baseline BCAP total score	12mo BCAP total score	Baseline BCAP risk cutoff (9+)	12mo BCAP risk cutoff (9+)	Mother’s ACEs	Household income bracket	Mother’s marital status	Mother’s education	Mother’s age	Mother’s race/ethnicity	M (SD), Mdn, or N (%)
*Treatment*													
Treatment Received (1+ session)	73												33 (45.20%)
Number of IMH-HV visits	**0.81**	73											11.77 (16.02)
*Child abuse potential*													
Baseline BCAP Total Score	−0.08	0.10	73										9.23 (5.24)
12mo BCAP Total Score	−0.26	−0.20	**0.64**	66									6.46 (5.13)
Baseline BCAP risk cutoff (9+)	−0.12	0.04	**0.82**	**0.50**	73								38 (52.10%)
12mo BCAP risk cutoff (9+)	−0.24	−0.15	**0.55**	**0.87**	**0.54**	66							23 (31.50%)
*Baseline sample characteristics*													
Mother’s ACEs	−0.08	−0.02	**0.35**	0.20	0.19	0.12	73						3.64 (2.40)
Household Income bracket	0.04	0.11	−0.20	−0.19	−0.10	−0.19	0.15	72					Mdn = $40,0000–$44,999/yr
Mother’s marital status	0.00	0.05	−0.26	−0.15	−0.09	−0.08	0.03	**0.53**	73				51 (69.9%)
Mother’s education	0.17	0.16	**−0.35**	−0.29	−0.27	−0.29	−0.18	**0.58**	**0.45**	73			Mdn = Bachelor’s degree
Mother’s age	−0.03	0.05	−0.23	−0.18	−0.07	−0.16	−0.04	**0.53**	**0.50**	**0.49**	73		31.91 (5.69)
Mother’s race/ethnicity (any non-white race or ethnicity endorsed)	−0.08	−0.11	0.21	0.24	0.07	0.12	0.09	**−0.57**	**−0.38**	**−0.32**	**−0.34**	73	32 (43.80%)

Bolded values indicate a medium or large effect size.

At baseline, independent samples t-tests and chi-square tests of independence revealed that participants who received at least one home visit had slightly higher educational attainment than those who did not receive any home visits, *t*(71) = −1.446, *p* = 0.076. There were no differences between these groups for any other study variables.

### Participation in IMH-HV treatment

We examined the association between treatment received (none vs. some) and 12-month child abuse potential scores with regression analysis. Controlling for baseline child abuse potential scores, participants who received any treatment had lower 12-month child abuse potential scores than those who received no treatment, with a small effect size, Δ*R*^2^ = 0.045, *β* = −0.213, *t* = −2.280, *p* = 0.026. There was no change in results when controlling for educational attainment.

### IMH-HV treatment dosage

**Figure 2:**
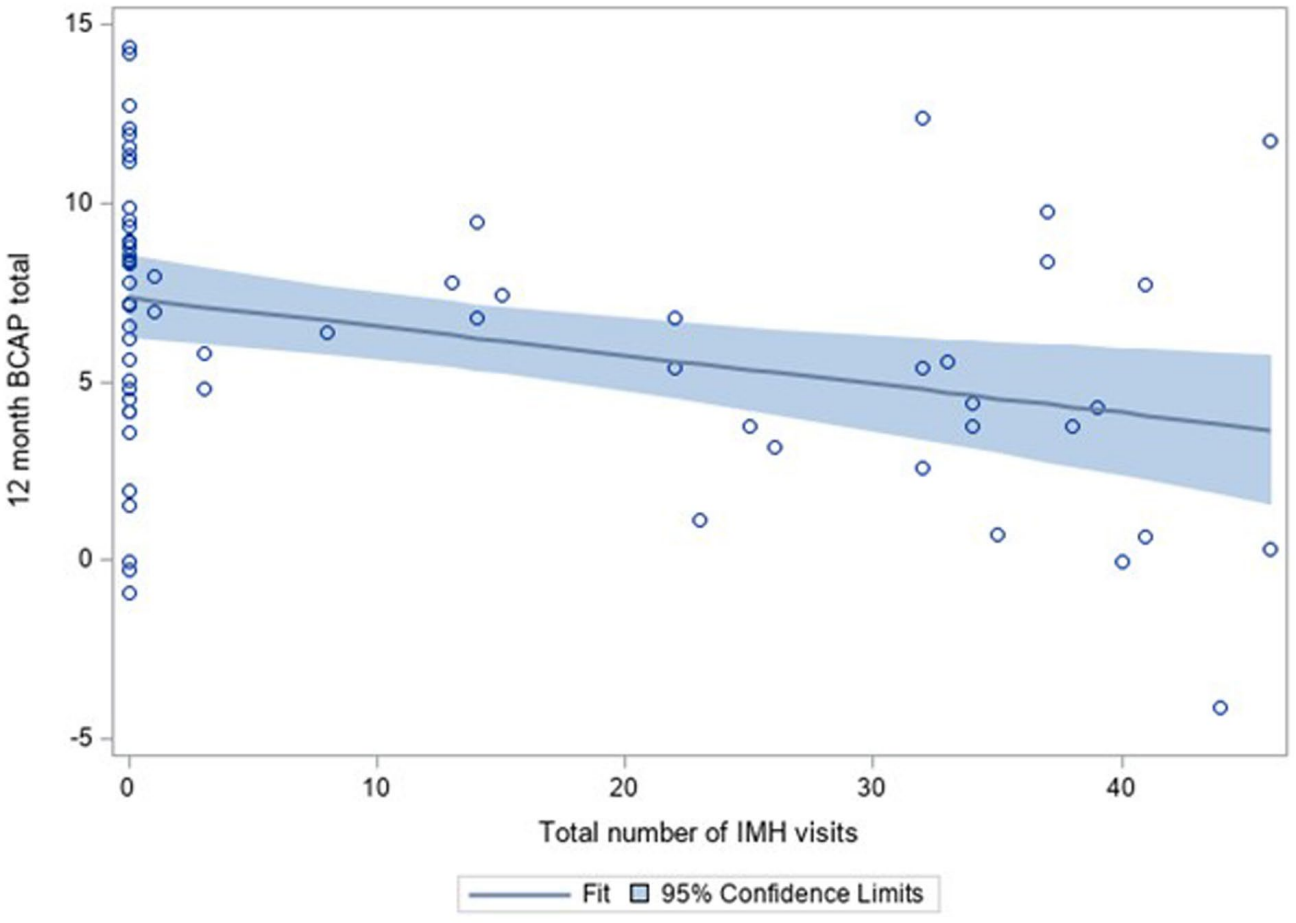
BCAP scores at 12 months by number of IMH home visits, controlling for baseline BCAP scores, including scatterplot and 95% confidence limits.

Dosage of IMH treatment was tested in regression analysis using the continuous measure of total number of IMH home visits as a predictor of 12-month child abuse potential. Controlling for baseline child abuse potential, results showed that more IMH home visits were associated with lower child abuse potential at 12 months, with a small effect size, Δ*R*^2^ = 0.07, *β* = −0.26, *t* = −2.84, *p* = 0.01 (Figure 2). There was no change in results when controlling for educational attainment.

### Child abuse potential risk

**Figure 3:**
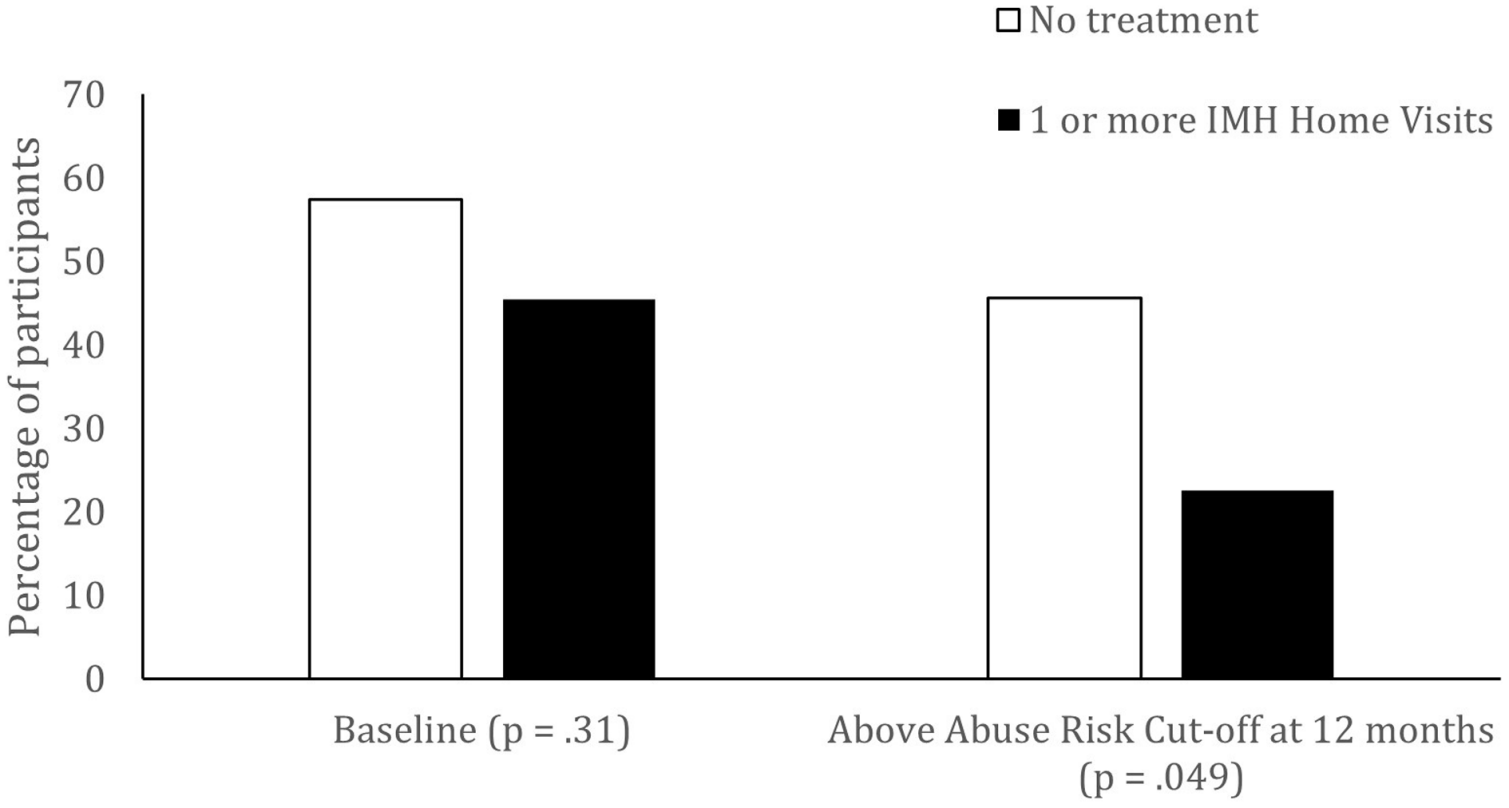
Participation in treatment in relation to child abuse risk categorization at 12 months.

Chi square tests were used to examine whether participation in treatment was related to participants scoring above a risk cutoff of 9 on the BCAP. At baseline, treatment group was not related to scoring above or below the risk range on the BCAP, *X*^2^ (1, *n* = 66) = 0.89, *p* = 0.34. However, at the 12-month time point, participants who received at least one treatment session were more likely to be below the at-risk range (77%) and those who received no treatment had similar numbers of participants above (45%) and below the risk range (54%), *X*^2^ (1, *n* = 66) = 3.88, *p* = 0.05 (Figure 3). In other words, participation in treatment appears to be related to a reduced likelihood of scoring in the risk range for child abuse potential. In the subsample of participants who had no invalid responses, results showed the same pattern, but with a slightly lower chi square value, *X*^2^ (1, *n* = 44) = 3.39, *p* = 0.07.

## Discussion

It is vital to find effective interventions to prevent child maltreatment and ameliorate the negative impact of early maltreatment, especially for infants and toddlers who are overrepresented in the child welfare system (30). In Michigan, 18% of children have had at least one report to CPS by third grade (4). The purpose of this paper was to replicate the previous finding that IMH-HV is effective at reducing risk for child maltreatment (23), using a more rigorous study design. Consistent with previous work, we found that participating in IMH-HV reduced the risk for child maltreatment and that mothers who had more IMH-HV treatment had significantly lower child abuse potential scores at 12 months.

IMH-HV may be effective at reducing risk for maltreatment because core components of the model address multiple risk factors that predict maltreatment. In the Michigan Model of IMH-HV (18), clinicians assist with material needs and support the parent in developing coping skills and social supports to address the stressors associated with poverty that can impact parenting. Importantly, IMH-HV clinicians build a trusting alliance with the parent that supports the delivery of infant-parent psychotherapy. While other home visiting programs also attend to the provider-parent relationship [e.g., (31, 32)], IMH-HV may include particularly helpful elements. IMH-HV clinicians engage parents in work together, a critical element in home visiting beyond parents’ satisfaction with their home visitors (33). Also, home visiting that is customized to each parent’s particular needs is related to parents’ sense of empowerment (34). Hence, IMH-HV is different from other home visiting models in the therapeutic attention that is paid to the developing parent-infant relationship and attending to the parents’ developmental history, including parents’ prior experiences of trauma. IMH clinicians are specifically trained to engage with and support parents in processing trauma and relationship disturbances and ruptures. As Fraiberg et al. (35) noted long ago, “ghosts in the nursery,” (i.e., unresolved childhood trauma) may disrupt the parents’ capacity to accurately appraise and respond to infant distress, resulting in an increased risk of child abuse. Additionally, previous research suggests that IMH-HV is effective at improving parental reflective functioning, which is foundational for regulating one’s own distress and contingently responding to children’s needs (25, 26). Such components that are unique to IMH-HV may be important in reducing maltreatment risk.

In addition to attending to concrete needs and providing infant-parent psychotherapy, clinicians offer emotional support as well as individualized developmental guidance unique to each infant. Furthermore, promoting parental mental health is a core focus in IMH-HV. Parents with psychological vulnerabilities, such as low mastery and depressive symptoms, may particularly benefit from home visiting relative to reduced maltreatment risk (36). The relationship-based nature of IMH-HV promotes parents’ mastery and psychological well-being (37), perhaps contributing to reduced risks for maltreatment. As home visiting is often not enough to address all of a family’s needs, a core goal of IMH-HV is to connect families with other needed services. This can include individual psychotherapy for a parent with mental health concerns, or connection to entitlement programs (e.g., WIC, cash assistance) for families experiencing financial insecurity. In fact, preliminary analyses suggest that children receiving IMH-HV receive more referrals to other service (e.g., referrals to the Early On program for developmental support) than children in the control group. It is possible that when families’ needs are met—both through IMH-HV and other programs and services—their risk factors for child maltreatment (e.g., emotional distress, social isolation, stress) may also decrease.

Another central component of the delivery of IMH-HV services is the use of parallel process (38). Parents served by this model are often lonely and isolated and many have few memories of positive relationships in their own childhood. As IMH-HV clinicians provide a holding environment that conveys to the parent a deep sense of care and respect, parents often experience a new kind of relationship that fuels their capacity to “hold and contain” their own infant. The respectful and professionally nurturing relationship with the IMH-HV clinician also helps the parent access “angels in the nursery” [i.e., memories of being loved, cared for, and protected; (39)] that serve to increase parental understanding of their own infants’ tender needs. By reducing emotional distress, reducing rigidly held beliefs about infant behavior, and increasing social support, IMH-HV services provide parents the relational experience that other home visiting interventions for maltreating families may not. IMH-HV services provided to families in the child welfare system are effective at improving parenting behavior (26), while standard parenting interventions may be less effective (40). Home visitors, too, experience a safe holding environment with their supervisors. Specifically, the provision of reflective supervision is a key element in IMH-HV. Just as home visiting clinicians provide an environment of holding and containing for parents to enable their responsiveness to their infants, so, too, do supervisors nurture clinicians, better enabling the complex work of home visiting. In fact, a meta-analysis (11) of home visiting intervention effects on maltreatment showed that receipt of reflective supervision was associated with more robust intervention effects.

While other home visiting models also focus on reducing parental stress, building parental knowledge of child development, and assisting families in accessing a variety of needed resources, the unique features of IMH-HV, namely a masters level clinician building a trusting alliance with the parent and providing infant-parent psychotherapy and emotional support, may be particularly helpful in reducing maltreatment risk. Home visiting models that focus more on the provision of support and increasing parents’ knowledge of development may not be intensive enough to interrupt intergenerational transmission of trauma.

### Strengths, limitations, and future directions

Study strengths include the experimental design, and high retention of participants over time in the study. While our methodology included randomized assignment of participants to IMH-HV or a control condition, we had a small sample size and 5 participants assigned to treatment who did not participate in any treatment sessions. Due to the intensive nature of the intervention, a larger sample size was not possible. Thus, intent to treat analysis was not possible, and instead participation in treatment was examined. Our study sample included mothers, but not fathers; while mothers represent the majority of IMH-HV participants in the community, it is not known whether results generalize to fathers or other caregivers (grandparents, foster parents, guardians) who participate in IMH-HV. Further, this study utilized a validated measure of child abuse potential that taps into factors that increase risk for child abuse. While data was not available to assess substantiated child abuse in this sample, this questionnaire measure captures a wide range of behaviors that are known to increase risk for maltreatment. Future research is needed to examine how various program components are specifically related to reduction in child maltreatment risk, and whether there are subpopulations that differ in their response to the intervention. Future studies should also compare IMH-HV to other evidence-based home visiting models to better determine which interventions work best for whom. While the current study did collect some measures up to 12 months after treatment had ended to assess the persistence of positive intervention effects over time, these later time points were confounded by the onset of the COVID-19 pandemic, limiting the interpretability of these data. Future studies of IMH-HV should examine the longer-term outcomes of IMH-HV, including whether parents were connected to other treatments or programs (e.g., parent psychotherapy) that may provide longer-term support to parents and families and further mitigating the risk for child maltreatment.

## Conclusion

IMH-HV is a promising model to reduce the likelihood of child maltreatment among families experiencing psychosocial risk. Our findings suggest that participation in any IMH-HV treatment, and especially participation in more sessions of treatment, reduces risk for child maltreatment. IMH-HV’s focus on assisting with material needs, supporting caregivers’ mental health and their parental reflective capacity, increasing social connections, and providing infant parent psychotherapy are likely to underlie the positive effects of this program.

## Data availability statement

The raw data supporting the conclusions of this article will be made available by the authors, without undue reservation.

## Ethics statement

The studies involving human participants were reviewed and approved by University of Michigan Medical School Institutional Review Board. The patients/participants provided their written informed consent to participate in this study.

## The Michigan Collaborative for Infant Mental Health Research members

The Michigan Collaborative for Infant Mental Health Research (MCIMHR) is composed of researchers from eight universities and from the Alliance for the Advancement of Infant Mental Health, each of whom has collaborated in the design and implementation of the current study. MCIMHR members include (in alphabetical order): Carla Barron, HB-H, Nora L. Erickson, Hiram E. Fitzgerald, Alissa C. Huth-Bocks, JJ, MJ, JL, Alyssa S. Meuwissen, Alison L. Miller, MM, Larissa N. Niec, JeR, JuR, KR, Sarah E. Shea, Paul Spicer, AS, Laurie Van Egeren, Christopher L. Watson, and Deborah J. Weatherston.

## Author contributions

MJ, JeR, KW, JL, HB-H, JuR, AS, and JJ each wrote portions of the first draft of the manuscript. JJ performed the statistical analysis. KR and MM contributed to conception, design, and funding acquisition for the broader study. All authors contributed to the manuscript revision, read, and approved the submitted version.

## Funding

This project was supported by funds from the Michigan Department of Health and Human Services, the Michigan Department of Health and Human Services Community Mental Health Services Block Grant, the Michigan Health Endowment Fund and the University of Michigan Department of Psychiatry’s Women and Infants Mental Health Program (PIs: KR and MM). HB-H’s efforts were partially funded by the USDA National Institute of Food and Agriculture Hatch project MIC02700.

## Conflict of interest

The authors declare that the research was conducted in the absence of any commercial or financial relationships that could be construed as a potential conflict of interest.

## Publisher’s note

All claims expressed in this article are solely those of the authors and do not necessarily represent those of their affiliated organizations, or those of the publisher, the editors and the reviewers. Any product that may be evaluated in this article, or claim that may be made by its manufacturer, is not guaranteed or endorsed by the publisher.

## References

[1] Jones Harden B Buhler A Parra LJ. Maltreatment in infancy: a developmental perspective on prevention and intervention. Trauma Violence Abuse. (2016) 17:366–86. doi: 10.1177/1524838016658878 27580663

[2] Li M D’Arcy C Meng X. Maltreatment in childhood substantially increases the risk of adult depression and anxiety in prospective cohort studies: systematic review, meta-analysis, and proportional attributable fractions. Psychol Med. (2016) 46:717–30. doi: 10.1017/S0033291715002743, PMID: 26708271

[3] Norman RE Byambaa M De R Butchart A Scott J Vos T. The long-term health consequences of child physical abuse, emotional abuse, and neglect: a systematic review and meta-analysis. PLoS Med. (2012) 9:e1001349. doi: 10.1371/journal.pmed.1001349, PMID: 23209385 PMC3507962

[4] Ryan JP Jacob BA Gross M Perron BE Moore A Ferguson S. Early exposure to child maltreatment and academic outcomes. Child Maltreat. (2018) 23:365–75. doi: 10.1177/1077559518786815, PMID: 30037281

[5] Capaldi D DeGarmo D Patterson GR Forgatch M. Contextual risk across the early life span and association with antisocial behavior In: Antisocial Behavior in Children and Adolescents: A Developmental Analysis and Model for Intervention. Washington: American Psychological Association (2002). 123–45.

[6] Doidge JC Higgins DJ Delfabbro P Edwards B Vassallo S Toumbourou JW. Economic predictors of child maltreatment in an Australian population-based birth cohort. Child Youth Serv Rev. (2017) 72:14–25. doi: 10.1016/j.childyouth.2016.10.012

[7] Widom CS Czaja SJ DuMont KA. Intergenerational transmission of child abuse and neglect: real or detection bias? Science. (2015) 347:1480–5. doi: 10.1126/science.1259917, PMID: 25814584 PMC5308058

[8] Doidge JC Higgins DJ Delfabbro P Segal L. Risk factors for child maltreatment in an Australian population-based birth cohort. Child Abuse Negl. (2017) 64:47–60. doi: 10.1016/j.chiabu.2016.12.002, PMID: 28027464

[9] Michalopoulos C Faucetta K Hill CJ Portilla XA Burrell L Lee H. Impacts on Family Outcomes of Evidence-Based Early Childhood Home Visiting: Results from the Mother and Infant Home Visiting Program Evaluation. Washington, DC: Office of Planning, Research, and Evaluation, Administration for Children and Families, U.S. Department of Health and Human Services (2019).

[10] Sama-Miller E Akers L Mraz-Esposito A Zukiewicz M Avellar S Paulsell D. Home Visiting Evidence of Effectiveness Review: Executive Summary. Washington, DC: Department of Health and Human Services (2016).

[11] Casillas KL Fauchier A Derkash BT Garrido EF. Implementation of evidence-based home visiting programs aimed at reducing child maltreatment: a meta-analytic review. Child Abuse Negl. (2016) 53:64–80. doi: 10.1016/j.chiabu.2015.10.009, PMID: 26724823

[12] Duggan A Caldera D Rodriguez K Burrell L Rohde C Crowne SS. Impact of a statewide home visiting program to prevent child abuse. Child Abuse Negl. (2007) 31:801–27. doi: 10.1016/j.chiabu.2006.06.011, PMID: 17822764

[13] Vaithianathan R Wilson M Maloney T Baird S. The Impact of the Family Start Home Visiting Programme on Outcomes for Mothers and Children: A Quasi-Experimental Study. Wellington, NZ: Ministry of Social Development (2016).

[14] Berthelot N Ensink K Bernazzani O Normandin L Luyten P Fonagy P. Intergenerational transmission of attachment in abused and neglected mothers: the role of trauma-specific reflective functioning. Infant Ment Health J. (2015) 36:200–12. doi: 10.1002/imhj.21499, PMID: 25694333

[15] Narayan AJ Ippen CG Harris WW Lieberman AF. Protective factors that buffer against the intergenerational transmission of trauma from mothers to young children: a replication study of angels in the nursery. Dev Psychopathol. (2019) 31:173–87. doi: 10.1017/S0954579418001530, PMID: 30757987

[16] Fraiberg S Adelson E Shapiro V. Ghosts in the nursery: a psychoanalytic approach to the problems of impaired infant-mother relationships In: Clinical Studies in Infant Mental Health. New York: Basic Books (1980). 164–96. 10.1016/s0002-7138(09)61442-4 1141566

[17] Lawler JM Rosenblum KL Muzik M Ludtke M Weatherston DJ Tableman B. A collaborative process for evaluating infant mental health home visiting in Michigan. Psychiatr Serv. (2017) 68:535–8. doi: 10.1176/appi.ps.201700047, PMID: 28412898

[18] Weatherston DJ Ribaudo J. The Michigan infant mental health home visiting model. Infant Ment Health J. (2020) 41:166–77. doi: 10.1002/imhj.21838, PMID: 32242955

[19] Weatherston DJ Tableman B. Infant Mental Health Home Visiting. Southgate, MI: Michigan Association for Infant Mental Health (2015).

[20] Huth-Bocks AC Jester JM Stacks AM Muzik M Rosenblum KL The Michigan Collaborative for Infant Mental Health Research. Infant mental health home visiting therapists’ fidelity to the Michigan IMH-HV model in community practice settings. Infant Ment Health J. (2020) 41:206–19. doi: 10.1002/imhj.21839 32242965

[21] Weatherston D. J. (2010). Infant mental health home visiting strategies. Zero to Three, July 2–7.

[22] Mountain G Cahill J Thorpe H. Sensitivity and attachment interventions in early childhood: a systemic review and meta-analysis. Infant Behav Dev. (2017) 46:14–32. doi: 10.1016/j.infbeh.2016.10.006, PMID: 27870988

[23] Julian MM Muzik M Jester JM Handelzalts J Erickson N Stringer M. Relationships heal: reducing harsh parenting and child abuse potential with relationship-based parent-infant home visiting. Child Youth Serv Rev. (2021) 128:106135. doi: 10.1016/j.childyouth.2021.106135

[24] Rosenblum KL Muzik M Jester JM Huth-Bocks A Erickson N Ludtke M. Community-delivered infant–parent psychotherapy improves maternal sensitive caregiving: evaluation of the Michigan model of infant mental health home visiting. Infant Ment Health J. (2020) 41:178–90. doi: 10.1002/imhj.21840, PMID: 32242953

[25] Stacks AM Jester JM Wong K Huth-Bocks A Brophy-Herb H Lawler J. Infant mental health home visiting: intervention dosage and therapist experience interact to support improvements in maternal reflective functioning. Attach Hum Develop. (2021) 24:53–75. doi: 10.1080/14616734.2020.1865606, PMID: 33427582

[26] Stacks AM Barron CC Wong K. Infant mental health home visiting in the context of an infant—toddler court team: changes in parental responsiveness and reflective functioning. Infant Ment Health J. (2019) 40:523–40. doi: 10.1002/imhj.21785, PMID: 31095763

[27] Stacks AM Wong K Barron C Ryznar T. Permanency and well-being outcomes for maltreated infants: pilot results from an infant-toddler court team. Child Abuse Negl. (2020) 101:104332. doi: 10.1016/j.chiabu.2019.104332, PMID: 31926458

[28] Ondersma SJ Chaffin MJ Mullins SM LeBreton JM. A brief form of the child abuse potential inventory: development and validation. J Clin Child Adolesc Psychol. (2005) 34:301–11. doi: 10.1207/s15374424jccp3402_9, PMID: 15901230

[29] Milner JS Gold RG Wimberley RC. Prediction and explanation of child abuse. Cross-validation of the child abuse potential inventory. J Consult Clin Psychol. (1986) 54:865–6. doi: 10.1037/0022-006X.54.6.865, PMID: 3794036

[30] U.S. Department of Health & Human Services Administration on Children, Youth and Families, Children’s Bureau (2021). Child Maltreatment 2019. Available at: https://www.acf.hhs.gov/cb/report/child-maltreatment-2019

[31] Kanda K Blythe S Grace R Kemp L. Parent satisfaction with sustained home visiting care for mothers and children: an integrative review. BMC Health Serv Res. (2022a) 22:295–14. doi: 10.1186/s12913-022-07666-3, PMID: 35241062 PMC8895511

[32] Korfmacher J Green B Staerkel F Peterson C Cook G Roggman L. Parent involvement in early childhood home visiting. Child Youth Care Forum. (2008) 37:171–96. doi: 10.1007/s10566-008-9057-3

[33] Staudt M. Treatment engagement with caregivers of at-risk children: gaps in research and conceptualization. J Child Fam Stud. (2007) 16:183–96. doi: 10.1007/s10826-006-9077-2

[34] Kanda K Blythe S Grace R Elcombe E Kemp L. Does customised care improve satisfaction and positively enable parents in sustained home visiting for mothers and children experiencing adversity? BMC Health Serv Res. (2022b) 22:1361. doi: 10.1186/s12913-022-08759-9, PMID: 36384551 PMC9670446

[35] Fraiberg S Adelson E Shapiro V. Ghosts in the nursery: a psychoanalytic approach to the problems of impaired infant-mother relationships. J Am Acad Child Psychiatry. (1975) 14:387–421. doi: 10.1016/S0002-7138(09)61442-4, PMID: 1141566

[36] DuMont K Mitchell-Herzfeld S Greene R Lee E Lowenfels A Rodriguez M. Healthy families New York (HFNY) randomized trial: effects on early child abuse and neglect. Child Abuse Negl. (2008) 32:295–315. doi: 10.1016/j.chiabu.2007.07.007, PMID: 18377991

[37] McKelvey L Schiffman RF Brophy-Herb HE Bocknek EL Fitzgerald HE Reischl TM. Examining long-term effects of an infant mental health home-based early head start program on family strengths and resilience. Infant Ment Health J. (2015) 36:353–65. doi: 10.1002/imhj.21518, PMID: 26118949

[38] Tomlin AM Viehweg SA. Infant mental health: making a difference. Prof Psychol Res Pract. (2003) 34:617–25. doi: 10.1037/0735-7028.34.6.617

[39] Lieberman AF Padrón E Van Horn P Harris WW. Angels in the nursery: the intergenerational transmission of benevolent parental influences. Infant Ment Health J. (2005) 26:504–20. doi: 10.1002/imhj.20071, PMID: 28682485

[40] Casanueva C Martin SL Runyan DK Barth RP Bradley RH. Parenting services for mothers involved with child protective services: do they change maternal parenting and spanking behaviors with young children? Child Youth Serv Rev. (2008) 30:861–78. doi: 10.1016/j.childyouth.2007.12.013

[41] Bakermans-Kranenburg MJ Van IJzendoorn MH Bradley RH. Those who have, receive: the Matthew effect in early childhood intervention in the home environment. Rev Educ Res. (2005) 75:1–26. doi: 10.3102/00346543075001001

[42] Kroenke K Spitzer RL. The PHQ-9: a new depression diagnostic and severity measure. Psychiatr Ann. (2002) 32:509–515.

[43] Felitti VJ Anda RF Nordenberg D Williamson DF Spitz AM Edwards V. Relationship of childhood abuse and household dysfunction to many of the leading causes of death in adults: The Adverse Childhood Experiences (ACE) Study. Am J Prev Med. (1998) 14:245–258. 9635069 10.1016/s0749-3797(98)00017-8

